# Anti- and Pro-Oxidant Properties of Essential Oils against Antimicrobial Resistance

**DOI:** 10.3390/antiox11091819

**Published:** 2022-09-15

**Authors:** Amanda Shen-Yee Kong, Sathiya Maran, Polly Soo-Xi Yap, Swee-Hua Erin Lim, Shun-Kai Yang, Wan-Hee Cheng, Yong-Hui Tan, Kok-Song Lai

**Affiliations:** 1 School of Biosciences, University of Nottingham Malaysia, Jalan Broga, Semenyih 43500, Selangor, Malaysia; 2 School of Pharmacy, Monash University Malaysia, Jalan Lagoon Selatan, Bandar Sunway 47500, Selangor Darul Ehsan, Malaysia; 3 Jeffrey Cheah School of Medicine and Health Sciences, Monash University Malaysia, Jalan Lagoon Selatan, Bandar Sunway 47500, Selangor Darul Ehsan, Malaysia; 4 Health Sciences Division, Abu Dhabi Women’s College, Higher Colleges of Technology, Abu Dhabi 41012, United Arab Emirates; 5 Faculty Health and Life Sciences, INTI International University, Persiaran Perdana BBN, Putra Nilai, Nilai 71800, Negeri Sembilan, Malaysia; 6 Department of Biotechnology, Faculty of Applied Sciences, UCSI University, UCSI Heights, 1, Jalan Puncak Menara Gading, Taman Connaught, Cheras, Wilayah Persekutuan Kuala Lumpur 56000, Malaysia

**Keywords:** pro-oxidant, antioxidant, antimicrobial resistance, essential oil, ROS

## Abstract

The rapid evolution of antimicrobial resistance (AMR) has remained a major public health issue, reducing the efficacy of antibiotics and increasing the difficulty of treating infections. The discovery of novel antimicrobial agents is urgently needed to overcome the challenges created by AMR. Natural products such as plant extracts and essential oils (EOs) have been viewed as potential candidates to combat AMR due to their complex chemistry that carries inherent pro-oxidant and antioxidant properties. EOs and their constituents that hold pro-oxidant properties can induce oxidative stress by producing reactive oxygen species (ROS), leading to biological damage in target cells. In contrast, the antioxidant properties scavenge free radicals through offsetting ROS. Both pro-oxidant and antioxidant activities in EOs represent a promising strategy to tackle AMR. Thus, this review aimed to discuss how pro-oxidants and antioxidants in EOs may contribute to the mitigation of AMR and provided a detailed description of the challenges and limitations of utilizing them as a means to combat AMR.

## 1. Introduction

Antimicrobial resistance (AMR) has caused significant detrimental effects on human health, contributing to increased mortality rates and infections due to the implementation of inefficacious antibiotic dosages [1]. According to the Centers for Disease Control and Prevention (CDC), human infections caused by antibiotic-resistant pathogens affect more than 2.8 million people, resulting in over 35,000 deaths annually in the United States [2]. The emergence of AMR is mainly due to the overprescription of antimicrobial agents by physicians and a lack of compliance by consumers [3]. Furthermore, easy accessibility and availability of antibiotics from over-the-counter pharmaceutical sales aggravate this issue. Due to the growth of industrialization, antibiotic residues discarded in environmental matrices such as water and soil contribute to environmental problems, and these substances were reported to be unstable in water [4]. Over the past three decades, AMR rates have risen considerably while the pipeline of new antibiotics has declined significantly [5]. The administration of treatments during infections in the clinical setting is becoming more complicated; it remains unclear if we will be able to treat common infections in the future. As such, there is a pressing need to better understand underlying resistance mechanisms and uncover novel therapeutic strategies to fight against AMR [6].

Oxidative stress is defined as the disturbance of the balance between reactive oxygen species (ROS) production and antioxidants to neutralize their harmful effects [7]. This imbalance leads to molecular cell damage and complex biochemical mechanisms are required to regulate the entire process [5]. ROS can be produced intracellularly in biological pathways or induced extracellularly by exogenous procedures [8]. Generally, traditional antibiotics induce ROS to cause secondary damage to the pathogens or exerting bactericidal effects as the main mechanism. To increase survivability, pathogens may employ different strategies such as increased enzyme production and formation of oxidant scavengers to avoid cellular damage caused by ROS. In this regard, ROS is suggested to be used in clinical practice based on its antimicrobial activity against a wide spectrum of pathogens, including multidrug-resistant organisms. In clinical studies, surgihoney reactive oxygen (SHRO) is the first ROS product that has shown great potency in controlling and eradicating bacterial bioburden and biofilm in chronic wounds, especially towards multidrug-resistant bacteria such as *Pseudomonas aeruginosa*, methicillin-resistant *Staphylococcus aureus* (MRSA), and vancomycin-resistant enterococci [9]. ROS can help to prevent and also to break down bacterial and fungal biofilms; these biofilms have remained a significant problem in many clinical settings by their increased resistance toward conventionally prescribed antimicrobials [10].

In addition to the promising outcomes with ROS therapy on infection models, researchers have diverted mining approaches towards medicinal plants, an option which seems to be more widely accepted by the public [7]. Natural products such as essential oil (EO) consisting of a plethora of chemical compounds are becoming a popular platform for researchers in drug discovery to improve antimicrobial efficacy and reduce the development of resistance [11]. Numerous studies have also demonstrated the efficacy of EOs from cinnamon (*Cinnamomum zeylanicum*), lemongrass (*Cymbopogon citratus*), tea tree (*Melaleuca alternifolia*), and rosemary (*Rosmarinus officinalis*) as promising antimicrobials [12,13,14,15,16]. Multiple studies have shown synergistic effects between various EOs and antibiotics, potentially providing a possible resolution to the antibiotic resistance issue in the clinical setting [1,17,18,19,20]. EOs have garnered great attention due to their accessibility, biocompatibility, and potential antibacterial capacities without inducing drug resistance [21]. In this review, we focused on EOs as oxidative stress inducers (as pro-oxidants and antioxidants), their mechanisms of action employed in AMR, as well as the potential utilization of ROS and EOs as a possibly effective solution to combat the development of AMR.

## 2. Sources and Functionality of Pro-Oxidant and Antioxidant

Pro-oxidants are chemicals that induce oxidative stress, either by generating ROS or by inhibiting antioxidant mechanisms [22]. ROS refers to reactive radicals including hydrogen peroxide, hydroxyl ion, hydroxyl radical, peroxide, singlet oxygen, and superoxide anion that are recognized as side products of some biological processes [8]. ROS may induce peroxidation in proteins and lipids as well as damage to nucleic acids [7]. Lipid peroxidation is a self-propagating chain reaction between ROS and membrane fatty acids which causes membrane damage and cell killing [23]. Interaction between ROS and proteins encourages covalent modification which destabilizes and inactivates protein. Furthermore, nucleic acid is also a common target of ROS, causing lesions and DNA breakage and leading to non-functional protein production that eventually kills the cells [24]. Antibiotics, for example, aminoglycosides, disrupt protein synthesis and produce large amounts of hydroxyl radicals. Thus, ROS inducers may serve as a potential therapeutic agent against antibiotic resistance.

Pathogens that produce ROS-detoxifying enzymes undergo several cellular, metabolic, and phenotypic changes in order to reduce cellular damage caused by the ROS when exposed to antibiotics [5]. Regulation of the genes involved in the bacterial defense response is complex but limited to regulators that can directly sense the levels of ROS and activate the gene transcription [25]. Two major global regulators, superoxide sensing SoxR and hydrogen peroxide sensing OxyR, play pivotal roles in gene regulation against ROS [26]. These transcription regulators contribute to the formation of biofilm, evasion of host immune responses, and antibiotic resistance via direct regulation of specific proteins.

On the other hand, antioxidants protect cells by a variety of mechanisms including the conversion of ROS to non-radical species, breakage of the oxidative chain reaction, and suppression of localized oxygen concentrations [27]. The general public today is more health-conscious and natural products are becoming a popular approach for researchers to undergo novel drug discoveries against AMR, considering their low side effects and cost-effectiveness [1]. EOs are a mixture of natural, volatile, and aromatic compounds extracted from medicinal plants through methods such as steam distillation, hydro distillation, and supercritical carbon dioxide in the form of secondary metabolites [28]. They exhibit potent antimicrobial properties against a wide spectrum of gram-positive and gram-negative bacteria with a lower likelihood of initiating multidrug resistance as compared with existing antibiotics [29].

The mechanism of antimicrobial action depends on the type of chemical constituents in the EOs. Mainly, lipophilic compounds in the EOs can easily penetrate the cell membrane of pathogens that play important roles in processes such as nutrient processing, structural protein-synthesizing, and energy production [30]. De Oliveira and colleagues (2022) demonstrated the ability of EO extracted from lemongrass in protecting erythrocytes against lipid peroxidation with high antioxidant activity when the medium was exposed to high levels of ROS [31]. Antioxidant properties of EOs are not only useful in fighting infections but also serve a role in the preservation of food from the toxic effects of oxidants [32]. The ability of EOs in modulating immune systems on interleukins and tumor necrosis factors increases the possibility of including them in the production of functional foods.

## 3. Mechanisms of EOs in Combating AMR by Inducing ROS

The killing of pathogens can be derived from the primary damage of antibiotics or a secondary lethal stress response mediated by ROS [33]. Bacteriostatic activity is related to initial lesion formation whereas bactericidal action may result from both primary lesions and the cellular response to primary damage [34]. Primary damage stimulates a pathway that leads to ROS accumulation and oxidative stress response as secondary damage when the primary damage is not severe enough to cause cell death directly. ROS acts as a potential solution to antibiotic resistance, emphasizing that it is not a replacement but could minimize the use of antibiotics to prevent further resistance.

Under aerobic conditions, antibiotic treatment generates ROS, which boosts the bactericidal effect and induces the production of ROS defense enzymes including catalase (CAT) and superoxide dismutase (SOD) [26]. There is much evidence of antibiotic-mediated ROS generation in *Escherichia coli*, *P. aeruginosa*, and *Acinetobacter baumannii* [35,36]. Polymyxin B is an example of bacterial antimicrobial peptides that were observed to induce rapid bacteria cell death through Fenton chemistry-mediated hydroxyl radical production in *A. baumannii* [37]. Similarly, superoxide and hydroxyl radical accumulation was detected in response to the treatment of *E. coli* with ampicillin and kanamycin [38]. This accumulation further amplified the oxidative damage and lethality of the antibiotics involved.

**Figure 1:**
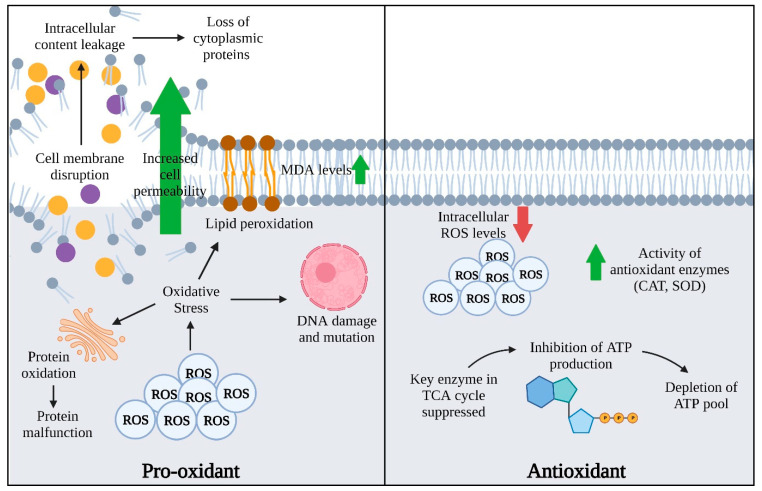
A schematic representation of pro-oxidant and antioxidant activities of EOs against clinically important pathogens. Both pro-oxidant and antioxidant properties of EOs act on different mechanisms of action that eventually lead to bacterial cell death.

EOs are a composite mixture of volatile compounds which possess not only antioxidant activities but also act as pro-oxidants as they affect the cellular redox status while contributing to cellular damage (Figure 1) [39]. Mainly, EO can be grouped into three main groups—terpenes, terpenoids, and aromatic compounds [1]. Yang and colleagues (2020) demonstrated the synergism effect of lavender (*Lavandula angustifolia*) EO and meropenem in treating carbapenemase-producing *Klebsiella pneumoniae* (KPC-KP) cells [19]. Comparative proteomic analysis revealed the presence of oxidative stress, suggesting that lavender EO and meropenem generated ROS in KPC-KP cells. Further validation tests confirmed the high concentrations of ROS and malondialdehyde (MDA), indicating the presence of lipid peroxidation and explaining the observed membrane disruption in KPC-KP cells.

More recently, Yang and colleagues (2021) revealed the accumulation of ROS and high levels of MDA in medium-treating KPC-KP cells with linalyl anthranilate (LNA), a compound that originated from the lavender plant [7]. This implies that the generation of ROS is induced and lipid peroxidation is triggered. With treatment of terpene LNA, the abundance of membrane-related proteins in treated cells was reduced, suggesting a disrupted bacterial membrane that led to intracellular leakage and loss of cytoplasmic proteins. LNA has a different approach to killing bacterial cells and their antibacterial activity is lower in terms of effective concentration as compared with commercialized antibiotics. The ability of LNA in inducing ROS may facilitate the uptake and enhance the activity of antibiotics.

Yang and colleagues (2019) demonstrated the antibacterial activity of Cinnamon bark (*Cinnamomum verum*) EO by inducing oxidative stress in KPC-KP cells [11]. Accumulation of ROS disrupted the bacterial membrane and enabled influx of ROS into the cells, leading to intracellular content leakage. Overproduction of ROS subsequently contributed to bacterial cell death by malfunctioning the proteins involved in energy production such as the adenosine triphosphate (ATP synthase), the electron transport complex, and the NADH-quinone oxidoreductases. The proteomic profile revealed the loss of three essential proteins involved in bacterial cell wall synthesis upon treatment with cinnamon bark EO. Deleterious effects were also observed in genetic damage and impairment of DNA and membrane repair systems. Exposure of cinnamon bark EO released the DNA mismatch repair protein (MutS) and DNA ligase, indicating the presence of damage in genetic materials of KPC-KP cells.

Brun and colleagues (2019) revealed oxidative damage in *C. glabrata* by the exposure of tea tree EO, indicating that ROS production is associated with the disruption in mitochondria membrane and organelles [40]. Incubation with tea tree EO also reduced biofilm formation in both gram-positive and gram-negative bacteria, with observed significant biofilm inhibition in MRSA and *P. aeruginosa*. The antiviral activity of tea tree EO is similar to its antibacterial effects in which both infectivity and uptake of Herpes simplex virus type 1 (HSV-1) were reduced upon administration of Tea Tree EO.

Khan and colleagues (2017) performed several analyses on the ascitic fluid of a patient with urinary tract infection to determine the mechanism action of carvacrol (phenolic monoterpenoid) against extended-spectrum beta-lactamase *E. coli* [41]. In the presence of carvacrol (MIC: 450 μg/mL), high generation of ROS and bacterial membrane depolarization were observed. Carvacrol induced the highly oxidative stress environment in the bacterial cell, changed the membrane permeability, and increased the leakage of cellular contents (DNA and proteins) by disrupting the bacterial membrane integrity. Further scanning electron microscopy analysis revealed induction of structural disruption by the interaction between carvacrol and the lipid bilayer of *E. coli*.

Similar findings were found in the study conducted by Kim and colleagues (2019). Thymol and carvacrol extracted from Thyme White (*Thymus vulgaris*) and Summer Savory (*Satureja hortensis*) EOs showed strong fumigant antifungal activity against *Raffaelea quercus-mongolicae* and *Rhizoctonia solani* [42]. Exposure of *R. quercus-mongolicae* and *R. solani* to thymol and carvacrol induced the production of ROS in two phytopathogenic fungi, disrupted fungal cell membranes, and contributed to fungal cell death. Interestingly, thymol showed higher antifungal activity than carvacrol in *R. quercus-mongolicae* but this effect was not significantly observed in *R. solani*.

Lee and colleagues (2020) experimented with different EOs on the same fungi species. *Trans*-cinnamaldehyde, neral, and geranial extracted from cinnamon bark and lemongrass EOs were associated with the induction of ROS production in *R. quercus-mongolicae* and *R. solani* [43]. Further microscopy analysis revealed the importance of the stated constituents in fungal cell membrane disruption, leading to fungal cell death. Table 1 summarizes the pro-oxidant activities of EOs appraised in this review.

## 4. Antioxidant Activities of EO in Mitigating AMR

EOs may act as pro-oxidants and antioxidants due to their complex chemistry [39]. Cinnamon bark EO, which was discussed in the previous section, was found to comprise 13 compounds, of which 4 were non-antioxidant compounds that are believed to be responsible for the induction of oxidative stress by generating ROS [11]. The presence of oxidative stress seems to conflict with the perception that the EO contains a high concentration of antioxidants.

Although earlier research has examined the antioxidant activity of EOs by free radical scavenging methods such as 2,2-diphenyl-1-picrylhydrazyl (DPPH) and 2,2′-azinobis-3-ethylbenzothiazoline-6-sulfonic acid (ABTS) assay [44,45], there is limited literature focusing on the mechanism of antioxidant action of EOs.

The outer membrane component lipopolysaccharide (LPS) of bacteria such as *E. coli* play a crucial role in its antimicrobial susceptibility [46]. Upon LPS binding, toll-like receptors on macrophages will activate the immune response’s downstream signaling pathways, including NFκB and MAPK [47]. In a review, de Lavor and colleagues (2018) highlighted the antioxidant activity of various EOs and their constituents in reducing ROS concentration, NF-Κb expression, and proinflammatory cytokines synthesis after the interaction with bacteria LPS [48]. Recent research by Pandur and colleagues (2022) revealed similar findings on the antioxidant capacity of thyme (*Thymus vulgaris* L.) EO and thymol [49]. Thyme EO and thymol significantly reduced the level of ROS at LPS-treated human macrophage cell lines after 6 h and 24 h. Increased levels of CAT and SOD activities were also observed, indicating the antioxidant properties of thyme EO and thymol.

Tang and colleagues (2021) employed metabolomics and found that the ROS concentrations were significantly lower in MRSA, while there was an increase in the concentration of *Amomum Villosum* EO compared with the control group [50]. *A. Villosum* EO inhibited the growth of MRSA by reducing the intracellular ROS levels, suggesting a different inhibition mechanism of bacterial growth compared with antibiotics. The activity of key enzymes involved in the tricarboxylic acid cycle was suppressed, leading to the inhibition of ATP production in MRSA. Hence, the reduction of ROS levels contributed to energy metabolic dysfunction and bacterial cell killing.

## 5. Challenges and Limitations in Combating AMR Using Plant-Derived Antimicrobials

In recent decades, there has been an emerging increase in the usage of EOs and their main constituents. The market for natural products is expected to grow substantially due to rising consumer demand. However, side effects of EOs such as allergic reactions and skin inflammation are present even when diluted [51,52]. Fuentes and colleagues (2021) showed the adverse effects of EOs and their components at medium and high concentrations exposure [53]. In vivo studies demonstrated adverse effects with acute and long-term usage of carvacrol and thymol in animals including mice, rats, and rabbits. Exposure to carvacrol, thymol, and eugenol caused skin irritation, inflammation, ulcer formation, dermatitis, and slow healing in human subjects. Interaction between the lipophilic property of EOs and the hydrophobic parts of the cell contributed to the toxicity mechanism [28]. Thus, more toxicological research focusing on chronic exposure and combined exposure is necessary to elucidate possible risks to the biological system for the preservation of human health.

Moreover, studies with plant-derived antimicrobials are lacking detail regarding the involved mechanisms. The antimicrobial mechanism of EO, for example, centralized on bacterial cell membrane disruption, which resulted in increased cell permeability and cell death. Several approaches including genomic profiling, proteomic profiling, transcriptomic, and metabolomics should be included in future research to provide a glimpse into cellular physiology and their mode of action with data combined from other approaches [1]. In addition, genetic changes of target microorganisms against EOs can be observed through comparative analysis of gene expression between EO-treated and untreated cells [54]. A panel of genes that are involved in pathogenic process, stress response, basic metabolism, and transcription regulation were identified when Kovács and colleagues (2019) employed quantitative real-time polymerase chain reaction to compare the activities of peppermint (*Mentha piperita*) EO in *Campylobacter jejuni* [55]. A high expression of oxidative stress response protein was also found in the study.

The metabolomics method is another potential tool for studying the antimicrobial mechanism of EO extracts using both quantitative and qualitative approaches [50]. Tang and colleagues (2021) identified key metabolic pathways that influenced MRSA activity as discussed in the previous section. Transcriptomic and proteomic profiling is becoming a popular approach that helps to measure gene expression and protein abundance [56]. Techniques such as two-dimensional polyacrylamide gel electrophoresis (2D-PAGE), stable isotopic labeling method, and matrix-assisted laser desorption/ionization time-of-flight mass spectrometry (MALDI-TOF MS) aid in finding the effective interactions of EO constituents with their targets, which are mostly proteins in nature [57]. Transcriptomic approaches can then be used to further validate the proteomics data by examining the gene expression profile and quantifying the protein abundance. With extensive knowledge of the mechanisms of plant-derived antimicrobials, development of novel antimicrobials for effective therapeutic usage that may help in reviving existing antibiotics is greatly anticipated in the near future.

## 6. Conclusions

In conclusion, both pro-oxidants and antioxidants bear promise as a starting point to tackle this global challenge of AMR. Future research should therefore focus on how pro-oxidants can benefit us without causing harm and the mechanism of action for current antioxidants to substitute and complement antibiotic therapies if needed. Discovery and development of multifaceted approaches that may effectively combat AMR are needed to balance off the rapid emergence of resistant pathogens. Both human and veterinary medicine must come up with a winning strategy by improving stewardship programs and identifying the best practices to prevent further antibiotic resistance, which is a truly One Health approach in AMR mitigation.

## Figures and Tables

**Table 1:** Recent insight of pro-oxidant activity of EOs against AMR.

Essential Oil/Essential Oil Constituents	Bacteria	Findings	References
Lavender	KPC-KP	Comparative proteomic analysis revealed the presence of oxidative stress, suggesting that Lavender EO and meropenem generated ROS in KPC-KP cells. High concentrations of ROS and MDA were detected, indicating the presence of lipid peroxidation, and explaining the observed membrane disruption in KPC-KP cells.	[19]
LNA	KPC-KP	Accumulation of ROS and high levels of MDA were detected, indicating the presence of lipid peroxidation. The abundance of membrane-related proteins in the treated cells was reduced, suggesting a disrupted bacterial membrane that led to intracellular leakage and loss of cytoplasmic proteins. the antibacterial activity of LNA is lower in terms of effective concentration as compared to commercialized antibiotics.	[7]
Cinnamon bark	KPC-KP	Oxidative stress was induced in KPC-KP cells. Accumulation of ROS disrupted the bacterial membrane and enabled the influx of ROS into the cells, leading to intracellular content leakage. Overproduction of ROS contributed to bacterial cell death by causing malfunction of the proteins involved in energy production such as the ATP synthase, the electron transport complex, and the NADH-quinone oxidoreductases. Proteomic profile revealed the loss of three essential proteins involved in bacterial cell wall synthesis. Deleterious effects were observed in genetic damage and impairment of DNA and membrane repair systems. Exposure of Cinnamon bark EO released the MutS and DNA ligase, indicating the presence of damage in the genetic materials of KPC-KP cells.	[11]
Tea Tree	*C. glabrata*, MRSA, HSV-1, *P. aeruginosa*	Oxidative damage was observed in *C. glabrata*, indicating that ROS production is associated with disruption in mitochondria membrane and organelles. Incubation with Tea Tree EO inhibited biofilm formation in MRSA and *P. aeruginosa.* The infectivity and uptake of HSV-1 were reduced upon administration of Tea Tree EO.	[40]
Carvacrol	Extended-spectrum beta-lactamase *E. coli*	Carvacrol induced a high production of ROS and bacterial membrane depolarization with its MIC value at 450 μg/mL The highly oxidative stress environment in the bacterial cell changed membrane permeability and increased the leakage of cellular contents (DNA and proteins) by disrupting the bacterial membrane integrity. Further scanning electron microscopy analysis revealed the induction of structural disruption by the interaction between carvacrol and the lipid bilayer of *E. coli*.	[41]
Thymol and Carvacrol	*R. quercus-mongolicae*, *R. solani*	Thymol and carvacrol showed strong fumigant antifungal activity against *R. quercus-mongolicae* and *R. solani*. Generation of ROS is induced and this disrupted the fungal cell membrane, contributing to fungal cell death. Thymol showed higher antifungal activity than carvacrol in *R. quercus-mongolicae* but this effect does not significantly observe in *R. solani*.	[42]
*Trans*-cinnamaldehyde, Neral, and Geranial	*R. quercus-mongolicae*, *R. solani*	*Trans*-cinnamaldehyde, neral, and geranial induced ROS production in *R. quercus-mongolicae* and *R. solani*. Further microscopy analysis revealed the importance of the stated constituents in fungal cell membrane disruption, leading to fungal cell death.	[43]

## References

[1] Yang S.-K., Low L.-Y., SOO-Xİ YAP P., Yusoff K., Mai C.-W., Lai K.-S., Erin Lim S.-H. (2018). Plant-derived antimicrobials: Insights into mitigation of antimicrobial resistance. Rec. Nat. Prod..

[2] CDC (2019). Antibiotic Resistance Threats in the United States. https://www.cdc.gov/drugresistance/about.html.

[3] Ramachandran P., Rachuri N.K., Martha S., Shakthivel R., Gundala A., Battu T.S. (2019). Implications of Overprescription of Antibiotics: A Cross-Sectional Study. J. Pharm. Bioallied Sci..

[4] Barreca S., Forni C., Colzani L., Clerici L., Daverio D., Dellavedova P. (2021). Study on the Stability of Antibiotics, Pesticides and Drugs in Water by Using a Straightforward Procedure Applying HPLC-Mass Spectrometric Determination for Analytical Purposes. Separations.

[5] Wanarska E., Mielko K.A., Maliszewska I., Młynarz P. (2022). The oxidative stress and metabolic response of Acinetobacter baumannii for aPDT multiple photosensitization. Sci. Rep..

[6] Dam S., Pagès J.M., Masi M. (2018). Stress responses, outer membrane permeability control and antimicrobial resistance in Enterobacteriaceae. Microbiology.

[7] Yang S.-K., Yusoff K., Ajat M., Yap W.-S., Lim S.-H.E., Lai K.-S. (2021). Antimicrobial activity and mode of action of terpene linalyl anthranilate against carbapenemase-producing Klebsiella pneumoniae. J. Pharm. Anal..

[8] Memar M.Y., Ghotaslou R., Samiei M., Adibkia K. (2018). Antimicrobial use of reactive oxygen therapy: Current insights. Infect. Drug Resist..

[9] Dryden M. (2017). Reactive oxygen therapy: A novel therapy in soft tissue infection. Curr. Opin. Infect. Dis..

[10] Dryden M. (2018). Reactive oxygen species: A novel antimicrobial. Int. J. Antimicrob. Agents.

[11] Yang S.-K., Yusoff K., Ajat M., Thomas W., Abushelaibi A., Akseer R., Lim S.-H.E., Lai K.-S. (2019). Disruption of KPC-producing Klebsiella pneumoniae membrane via induction of oxidative stress by cinnamon bark (*Cinnamomum verum* J. Presl) essential oil. PLoS ONE.

[12] Abd El-Aziz N.K., Ammar A.M., El-Naenaeey E.-S.Y.M., El Damaty H.M., Elazazy A.A., Hefny A.A., Shaker A., Eldesoukey I.E. (2021). Antimicrobial and antibiofilm potentials of cinnamon oil and silver nanoparticles against Streptococcus agalactiae isolated from bovine mastitis: New avenues for countering resistance. BMC Vet. Res..

[13] Chraibi M., Farah A., Elamin O., Iraqui H.M., Fikri-Benbrahim K. (2020). Characterization, antioxidant, antimycobacterial, antimicrobial effcts of Moroccan rosemary essential oil, and its synergistic antimicrobial potential with carvacrol. J. Adv. Pharm. Technol. Res..

[14] Lagha R., Ben Abdallah F., Al-Sarhan B.O., Al-Sodany Y. (2019). Antibacterial and Biofilm Inhibitory Activity of Medicinal Plant Essential Oils Against Escherichia coli Isolated from UTI Patients. Molecules.

[15] Elcocks E.R., Spencer-Phillips P.T.N., Adukwu E.C. (2020). Rapid bactericidal effect of cinnamon bark essential oil against Pseudomonas aeruginosa. J. Appl. Microbiol..

[16] Taleb M.H., Abdeltawab N.F., Shamma R.N., Abdelgayed S.S., Mohamed S.S., Farag M.A., Ramadan M.A. (2018). Origanum vulgare L. Essential Oil as a Potential Anti-Acne Topical Nanoemulsion-In Vitro and In Vivo Study. Molecules.

[17] Jesus G.S., Micheletti A.C., Takahashi K.M., Matayoshi T., Pott A., Yoshida N.C. (2020). Antimicrobial potential of Pectis substriata essential oil (Asteraceae) against drug-resistant Staphylococcus strains. An. Acad. Bras. Cienc..

[18] Yang S.-K., Yap P.S.-X., Krishnan T., Yusoff K., Kok-Gan C., Yap W.-S., Kok-Song L., Swee-Hua E.L. (2018). Mode of Action: Synergistic Interaction of Peppermint (*Mentha x piperita* L. Carl) Essential Oil and Meropenem Against Plasmid-Mediated Resistant E. col Shun-Kai Yang, Polly Soo-Xi Yap, Thiba Krishnan, Khatijah Yusoff, Kok-Gan Chan, Wai-Sum Yap, Kok-Song Lai and Swee-Hua Erin Lim. Rec. Nat. Prod..

[19] Yang S.-K., Yusoff K., Thomas W., Akseer R., Alhosani M.S., Abushelaibi A., Lim S.-H.-E., Lai K.-S. (2020). Lavender essential oil induces oxidative stress which modifies the bacterial membrane permeability of carbapenemase producing Klebsiella pneumoniae. Sci. Rep..

[20] Nafis A., Hassani L., Marraiki N., Al-Rashed S., Elgorban A.M., Syed A., Iriti M. (2021). Antimicrobial and synergistic effect of Moroccan native Argania spinosa essential oil for modulating of antibiotics resistance. Nat. Prod. Res..

[21] Wang Z., Bai H., Lu C., Hou C., Qiu Y., Zhang P., Duan J., Mu H. (2019). Light controllable chitosan micelles with ROS generation and essential oil release for the treatment of bacterial biofilm. Carbohydr. Polym..

[22] Sotler R., Poljšak B., Dahmane R., Jukić T., Pavan Jukić D., Rotim C., Trebše P., Starc A. (2019). Prooxidant Activities of Antioxidants and Their Impact on Health. Acta Clin. Croat..

[23] Su L.J., Zhang J.H., Gomez H., Murugan R., Hong X., Xu D., Jiang F., Peng Z.Y. (2019). Reactive Oxygen Species-Induced Lipid Peroxidation in Apoptosis, Autophagy, and Ferroptosis. Oxidative Med. Cell. Longev..

[24] Juan C.A., Pérez de la Lastra J.M., Plou F.J., Pérez-Lebeña E. (2021). The Chemistry of Reactive Oxygen Species (ROS) Revisited: Outlining Their Role in Biological Macromolecules (DNA, Lipids and Proteins) and Induced Pathologies. Int. J. Mol. Sci..

[25] Kashef N., Hamblin M.R. (2017). Can microbial cells develop resistance to oxidative stress in antimicrobial photodynamic inactivation?. Drug Resist. Updates.

[26] Shin B., Park C., Park W. (2020). Stress responses linked to antimicrobial resistance in Acinetobacter species. Appl. Microbiol. Biotechnol..

[27] Lourenço S.C., Moldão-Martins M., Alves V.D. (2019). Antioxidants of Natural Plant Origins: From Sources to Food Industry Applications. Molecules.

[28] Yap P.S., Yusoff K., Lim S.-H.E., Chong C.-M., Lai K.-S. (2021). Membrane Disruption Properties of Essential Oils—A Double-Edged Sword?. Processes.

[29] Li H., Yang T., Li F.-Y., Yao Y., Sun Z.-M. (2014). Antibacterial activity and mechanism of action of Monarda punctata essential oil and its main components against common bacterial pathogens in respiratory tract. Int. J. Clin. Exp. Pathol..

[30] Ramsey J.T., Shropshire B.C., Nagy T.R., Chambers K.D., Li Y., Korach K.S. (2020). Essential Oils and Health. Yale J. Biol. Med..

[31] De Oliveira E.S.F., Soares J.C.M., Valdez A., da Silva Ferreira M.V., da Silva Cecim M. (2022). Cymbopogon citratus Protects Erythrocytes from Lipid Peroxidation in vitro. Cardiovasc. Hematol. Agents Med. Chem..

[32] Valdivieso-Ugarte M., Gomez-Llorente C., Plaza-Díaz J., Gil Á. (2019). Antimicrobial, Antioxidant, and Immunomodulatory Properties of Essential Oils: A Systematic Review. Nutrients.

[33] Li H., Zhou X., Huang Y., Liao B., Cheng L., Ren B. (2021). Reactive Oxygen Species in Pathogen Clearance: The Killing Mechanisms, the Adaption Response, and the Side Effects. Front. Microbiol..

[34] Lam P.L., Wong R.S., Lam K.H., Hung L.K., Wong M.M., Yung L.H., Ho Y.W., Wong W.Y., Hau D.K., Gambari R. (2020). The role of reactive oxygen species in the biological activity of antimicrobial agents: An updated mini review. Chem. Biol. Interact..

[35] Kim S.Y., Park C., Jang H.J., Kim B.O., Bae H.W., Chung I.Y., Kim E.S., Cho Y.H. (2019). Antibacterial strategies inspired by the oxidative stress and response networks. J. Microbiol..

[36] Vaishampayan A., Grohmann E. (2021). Antimicrobials Functioning through ROS-Mediated Mechanisms: Current Insights. Microorganisms.

[37] Sampson T.R., Liu X., Schroeder M.R., Kraft C.S., Burd E.M., Weiss D.S. (2012). Rapid killing of Acinetobacter baumannii by polymyxins is mediated by a hydroxyl radical death pathway. Antimicrob. Agents Chemother..

[38] Wang X., Zhao X. (2009). Contribution of oxidative damage to antimicrobial lethality. Antimicrob. Agents Chemother..

[39] Mimica-Dukić N., Orčić D., Lesjak M., Šibul F. (2016). Essential Oils as Powerful Antioxidants: Misconception or Scientific Fact?. Medicinal and Aromatic Crops: Production, Phytochemistry, and Utilization.

[40] Brun P., Bernabè G., Filippini R., Piovan A. (2019). In Vitro Antimicrobial Activities of Commercially Available Tea Tree (*Melaleuca alternifolia*) Essential Oils. Curr. Microbiol..

[41] Khan I., Bahuguna A., Kumar P., Bajpai V.K., Kang S.C. (2017). Antimicrobial Potential of Carvacrol against Uropathogenic Escherichia coli via Membrane Disruption, Depolarization, and Reactive Oxygen Species Generation. Front. Microbiol..

[42] Kim J.-E., Lee J.-E., Huh M.-J., Lee S.-C., Seo S.-M., Kwon J.H., Park I.-K. (2019). Fumigant Antifungal Activity via Reactive Oxygen Species of Thymus vulgaris and Satureja hortensis Essential Oils and Constituents against Raffaelea quercus-mongolicae and Rhizoctonia solani. Biomolecules.

[43] Lee J.E., Seo S.M., Huh M.J., Lee S.C., Park I.K. (2020). Reactive oxygen species mediated-antifungal activity of cinnamon bark (*Cinnamomum verum*) and lemongrass (*Cymbopogon citratus*) essential oils and their constituents against two phytopathogenic fungi. Pestic. Biochem. Physiol..

[44] Radünz M., da Trindade M.L.M., Camargo T.M., Radünz A.L., Borges C.D., Gandra E.A., Helbig E. (2019). Antimicrobial and antioxidant activity of unencapsulated and encapsulated clove (*Syzygium aromaticum*, L.) essential oil. Food Chem..

[45] Torres-Martínez R., García-Rodríguez Y.M., Rios-Chavez P., Saavedra-Molina A., López-Meza J., Ochoa-Zarzosa A., Salgado-Garciglia R. (2018). Antioxidant Activity of the Essential Oil and its Major Terpenes of Satureja macrostema (Moc. and Sessé ex Benth.) Briq. Pharmacogn. Mag..

[46] Ebbensgaard A., Mordhorst H., Aarestrup F.M., Hansen E.B. (2018). The Role of Outer Membrane Proteins and Lipopolysaccharides for the Sensitivity of Escherichia coli to Antimicrobial Peptides. Front. Microbiol..

[47] Behzadi P., García-Perdomo H.A., Karpiński T.M. (2021). Toll-Like Receptors: General Molecular and Structural Biology. J. Immunol. Res..

[48] de Lavor É.M., Fernandes A.W.C., de Andrade Teles R.B., Leal A.E.B.P., de Oliveira Júnior R.G., Gama e Silva M., de Oliveira A.P., Silva J.C., de Moura Fontes Araújo M.T., Coutinho H.D.M. (2018). Essential Oils and Their Major Compounds in the Treatment of Chronic Inflammation: A Review of Antioxidant Potential in Preclinical Studies and Molecular Mechanisms. Oxidative Med. Cell. Longev..

[49] Pandur E., Micalizzi G., Mondello L., Horváth A., Sipos K., Horváth G. (2022). Antioxidant and Anti-Inflammatory Effects of Thyme (*Thymus vulgaris* L.) Essential Oils Prepared at Different Plant Phenophases on Pseudomonas aeruginosa LPS-Activated THP-1 Macrophages. Antioxidants.

[50] Tang C., Chen J., Zhou Y., Ding P., He G., Zhang L., Zhao Z., Yang D. (2021). Exploring antimicrobial mechanism of essential oil of Amomum villosum Lour through metabolomics based on gas chromatography-mass spectrometry in methicillin-resistant Staphylococcus aureus. Microbiol. Res..

[51] Das S., Horváth B., Šafranko S., Jokić S., Széchenyi A., Kőszegi T. (2019). Antimicrobial Activity of Chamomile Essential Oil: Effect of Different Formulations. Molecules.

[52] Salehi B., Mishra A.P., Shukla I., Sharifi-Rad M., Contreras M.D.M., Segura-Carretero A., Fathi H., Nasrabadi N.N., Kobarfard F., Sharifi-Rad J. (2018). Thymol, thyme, and other plant sources: Health and potential uses. Phytother. Res..

[53] Fuentes C., Fuentes A., Barat J.M., Ruiz M.J. (2021). Relevant essential oil components: A minireview on increasing applications and potential toxicity. Toxicol. Mech. Methods.

[54] Yang S.K., Tan N.P., Chong C.W., Abushelaibi A., Lim S.H., Lai K.S. (2021). The Missing Piece: Recent Approaches Investigating the Antimicrobial Mode of Action of Essential Oils. Evol. Bioinform. Online.

[55] Kovács J.K., Felső P., Horváth G., Schmidt J., Dorn Á., Ábrahám H., Cox A., Márk L., Emődy L., Kovács T. (2019). Stress Response and Virulence Potential Modulating Effect of Peppermint Essential Oil in Campylobacter jejuni. Biomed. Res. Int..

[56] Zhang C., Deng Y., Zhang G., Li J., Xiao A., Zhao L., Chen A., Tang H., Chang L., Pan G. (2022). Comparative Transcriptome and Proteome Analysis Provides New Insights Into the Mechanism of Protein Synthesis in Kenaf (*Hibiscus cannabinus* L.) Leaves. Front. Plant Sci..

[57] Aljaafari M.N., AlAli A.O., Baqais L., Alqubaisy M., AlAli M., Molouki A., Ong-Abdullah J., Abushelaibi A., Lai K.-S., Lim S.-H.E. (2021). An Overview of the Potential Therapeutic Applications of Essential Oils. Molecules.

